# Proteomic and functional analysis of HDL subclasses in humans and rats: a proof-of-concept study

**DOI:** 10.1186/s12944-023-01829-9

**Published:** 2023-06-29

**Authors:** Canxia Huang, Jie Zhang, Jingjing Huang, Hongwei Li, Kexin Wen, Jinlan Bao, Xiaoying Wu, Runlu Sun, Ayiguli Abudukeremu, Yue Wang, Zhijian He, Qiaofei Chen, Xinyi Huang, Hong Wang, Yuling Zhang

**Affiliations:** 1 Guangdong Province Key Laboratory of Arrhythmia and Electrophysiology, Guangzhou, 510120 China; 2 Critical Care Medicine Department, Sun Yat-Sen Memorial Hospital, Sun Yat-Sen University, Guangzhou, 510120 China; 3 Department of Cardiology, Sun Yat-Sen Memorial Hospital, Sun Yat-Sen University, Guangzhou, 510120 China; 4 Comprehensive Department, Sun Yat-Sen Memorial Hospital, Sun Yat-Sen University, Guangzhou, 510120 China; 5 Centers for Metabolic & Cardiovascular Research, Department of Pharmacology, Temple University School of Medicine, Philadelphia, PA 19140 USA

**Keywords:** High density lipoprotein, HDL subclass, Proteomics, Antioxidation, Cholesterol efflux, Fast protein liquid chromatography

## Abstract

**Background:**

The previous study investigated whether the functions of small, medium, and large high density lipoprotein (S/M/L-HDL) are correlated with protein changes in mice. Herein, the proteomic and functional analyses of high density lipoprotein (HDL) subclasses were performed in humans and rats.

**Methods:**

After purifying S/M/L-HDL subclasses from healthy humans (*n* = 6) and rats (*n* = 3) using fast protein liquid chromatography (FPLC) with calcium silica hydrate (CSH) resin, the proteomic analysis by mass spectrometry was conducted, as well as the capacities of cholesterol efflux and antioxidation was measured.

**Results:**

Of the 120 and 106 HDL proteins identified, 85 and 68 proteins were significantly changed in concentration among the S/M/L-HDL subclasses in humans and rats, respectively. Interestingly, it was found that the relatively abundant proteins in the small HDL (S-HDL) and large HDL (L-HDL) subclasses did not overlap, both in humans and in rats. Next, by searching for the biological functions of the relatively abundant proteins in the HDL subclasses via Gene Ontology, it was displayed that the relatively abundant proteins involved in lipid metabolism and antioxidation were enriched more in the medium HDL (M-HDL) subclass than in the S/L-HDL subclasses in humans, whereas in rats, the relatively abundant proteins associated with lipid metabolism and anti-oxidation were enriched in M/L-HDL and S/M-HDL, respectively. Finally, it was confirmed that M-HDL and L-HDL had the highest cholesterol efflux capacity among the three HDL subclasses in humans and rats, respectively; moreover, M-HDL exhibited higher antioxidative capacity than S-HDL in both humans and rats.

**Conclusions:**

The S-HDL and L-HDL subclasses are likely to have different proteomic components during HDL maturation, and results from the proteomics-based comparison of the HDL subclasses may explain the associated differences in function.

**Supplementary Information:**

The online version contains supplementary material available at 10.1186/s12944-023-01829-9.

## Background

The low level of high-density lipoprotein cholesterol (HDL-C) is correlated with the development of atherosclerosis and subsequent cardiovascular disease. However, some clinical trials [1, 2] have revealed that medications can raise HDL-C levels robustly, but these medications have off-target effects. A key potential reason is that directly targeting HDL-C could not take account of the complexity of high-density lipoprotein (HDL) subclasses, including their heterogeneous structure, composition and size. There are molar differences in the counts of the lipids and proteins in HDL, namely, apolipoproteins, cholesterol, sphingomyelin, phosphatidylcholine, and cholesteryl esters, and these molecules can account for the considerable heterogeneity in the shape, size, and charge of HDL [3]. Nevertheless, there are also some HDL-associated proteins function ranging from immunity, lipid transport, coagulation, antioxidation, complement regulation,, and metal ion transport. Recently, better than the concentration of HDL-C [4], bioassays have been developed to discover biomarkers that reflect the HDL function by proteomics and lipidomics [5]. These experiments identified a much more complex structure as follows: in addition to many kinds of lipids, HDL carries more than 80 different proteins [6] and even microRNAs (miRNAs) [7, 8]. Thus, the different HDL cargo, identified using proteomics, are good biomarkers that reflect the modulation of HDL functions. For example, O'Reilly, M. [9] found that the upregulations of acute-phase proteins (hemopexin (HPX), haptoglobin (HP), and serum amyloid A (SAA)) enriched on small HDL (S-HDL), but not efflux capacity, were novel biomarkers of impaired liver-to feces cholesterol excretion after intervention with saturated fat in mice.

HDL is commonly classified into different subclasses according to their density or size during maturation. Each of the HDL subclasses has unique chemical and biological properties. An increasing amount of studies have investigated the role of HDL subclass and their associated proteomic changes in determining their specific functions in human and mouse studies. For example, Martin, Seth [10] found that as a secondary intervention for coronary artery disease, low HDL3-C is associated with unfavorable clinical outcomes, but not HDL-C or HDL2-C, which highlights the benefits of subclassifying HDL-C. Dr. Heinecke [11] found that diabetes impairs the ATP-binding cassette transporter A1 (ABCA1)-mediated cholesterol efflux to S-HDL subclass by altering the levels of α1-antitrypsin (SERPINA1) and apolipoprotein C-II (APOC2) in the S-HDL subclass, and Dr. Vaisar Tomas [12] discovered that elevated levels of medium HDL (M-HDL) and paraoxonase(PON)-1 (PON1) could protect against diabetes-related vascular complications, independent of HDL-C concentrations. Because HDL is heterogeneous in composition, size and function during HDL maturation, it is essential to investigate the proteomic changes and functions related to HDL subclasses, and not only focus on identifying HDL subclasses.

Rats and mice have been used widely in mechanistic researches of HDL and its link to diseases. Many studies investigate HDL metabolism and function through treating rats with substances for disease models, such as for hyperlipidemia and diabetes models. However, there are significant differences in the lipid metabolism when comparing rats and humans. For example, in contrast to humans, rats carry cholesterol mainly in HDL particles due to low level of cholesteryl ester transfer protein (CETP), while CETP promotes transferring cholesterol esters from HDL to very low-density lipoprotein/low-density lipoprotein (LDL), and the low level of CETP in rats may explain why rats never develop atherosclerosis. Moreover, there is an overlap in LDL and HDL particles in rats, as shown by Dr. Lehmann [13], in which specific lymph and plasma HDL (isolated by ultracentrifugation) were analyzed for protein and lipid composition in Sprague–Dawley (SD) rats [14]. And recently, Dr. Boyan Liu [15] also found that hydrogen influences HDL-associated enzymes by performing proteomic analysis on rats fed a high-fat diet. Although both of these studies take proteins into consideration, there are no detailed studies focusing on the proteomic analysis of different HDL subclasses in rats.

Previous studies have indicated that to include samples with diverse HDL particle sizes and compositions, it is better purify the proteins by fast protein liquid chromatography (FPLC), rather than with ultracentrifugation, for proteomic analysis to identify unique proteins within the HDL subclasses [16, 17]. This proof-of-concept study was aiming to characterize the proteomic and functional differences in the HDL subclasses purified by FPLC from rat and healthy human samples.

## Methods

### Plasma collection from humans and rats

Male SD rats were used in this study. The animal protocol was in accordance with the institutional animal care and use committee of Sun Yat-Sen University. SD Rats were housed in a controlled environment and were fed during the day and fasted overnight. Rats were sacrificed after 10 weeks, and the venous blood were collected. The human protocol was registered at Chinese Clinical trial Registry (No. ChiCTR2000038859), and informed consent was obtained. Venous bloods were collected from 6 apparently healthy young males who had an overnight fast in ethylene diamine tetraacetic acid (EDTA)-coated tubes. The plasma was separated after centrifuging at ∼1590 × g for 15 min in a centrifuge (Eppendorf, centrifuge 5418R) and placed at 4 °C with the proper concentration of protease inhibitor within 16 h until gel filtration separation.

### Small, medium, and large HDL (S/M/L-HDL) subclasses isolated by FPLC

HDL was fractionated by FPLC as described previously [16]. Briefly, 500 μL of pooled plasma was added to three Superdex 200 Increase gel filtration columns (10/300 GL, GE Healthcare, Pittsburgh, PA) in an ÄKTA FPLC system (AKTA Avant 150, GE Healthcare, Pittsburgh, PA). The plasma was processed at 0.3 mL/min (system rate) in the Tris buffer containing 1 mM EDTA, 0.15 M NaCl, and 10 mM Tris. Eluates were acquired as FPLC fractions on the collector, and the HDL fractions were pooled evenly into S/M/L-HDL subclasses.

### Purification of HDL subclasses through binding phospholipids (PLs)

The collected equal volumes of HDL subclasses were further purified for PLs by a procedure of calcium silicate hydrate (CSH) resin (Supelco, Merk, US), which can tightly bind lipids and lipoproteins. CSH resin was developed to remove the lipids as described previously [18]. Briefly, 13.5 uL of CSH (100 mg/ml) was added to 600 μL of HDL subclass in a minicentrifuge (Fisher), which was mixed softly for 30 min and centrifugated (∼2200 × g for 2 min) at room temperature. The centrifugated CSH deposits were rinsed with 50 mM ammonium bicarbonate.

### Mass spectrometry

To obtain an unbiased assessment of all the proteins in the three subclasses, proteomics was conducted through liquid chromatography‒mass spectrometry/mass spectrometry (LC–MS/MS), which was conducted according to the previous study [16]. In brief, the CSH deposits of HDL subclass were underwent digestion with trypsin, followed by reduced with 5 mM dithiothreitol for 30 min and alkylated with 11 mM iodoacetamide for 15 min, and was next digested twice to obtain peptides. A equal volume (1.2 ml) of the digested peptides was degraded through high pH reversed-phase high-performance liquid chromatography, and then were subjected to an analytical column of an EASY-nLC1000 system.

The peptides were ionized by loading onto a nanospray ionization source, and then were detected by tandem mass spectrometry through a Thermo Scientific Q Exactive™ Plus connected online to ultra-performance liquid chromatography system. After the full scan and setting different Orbitrap resolution, the intact peptides and fragments were detected, and the data-dependent acquisition method was applied. The search engine of MaxQuant (version 1.5.2.8) was applied to process the mass spectrometry (MS) data, which were then analyzed based on the label-free quantification(LFQ).

### Identification of HDL proteins in human and rat samples

**Fig. 1:**
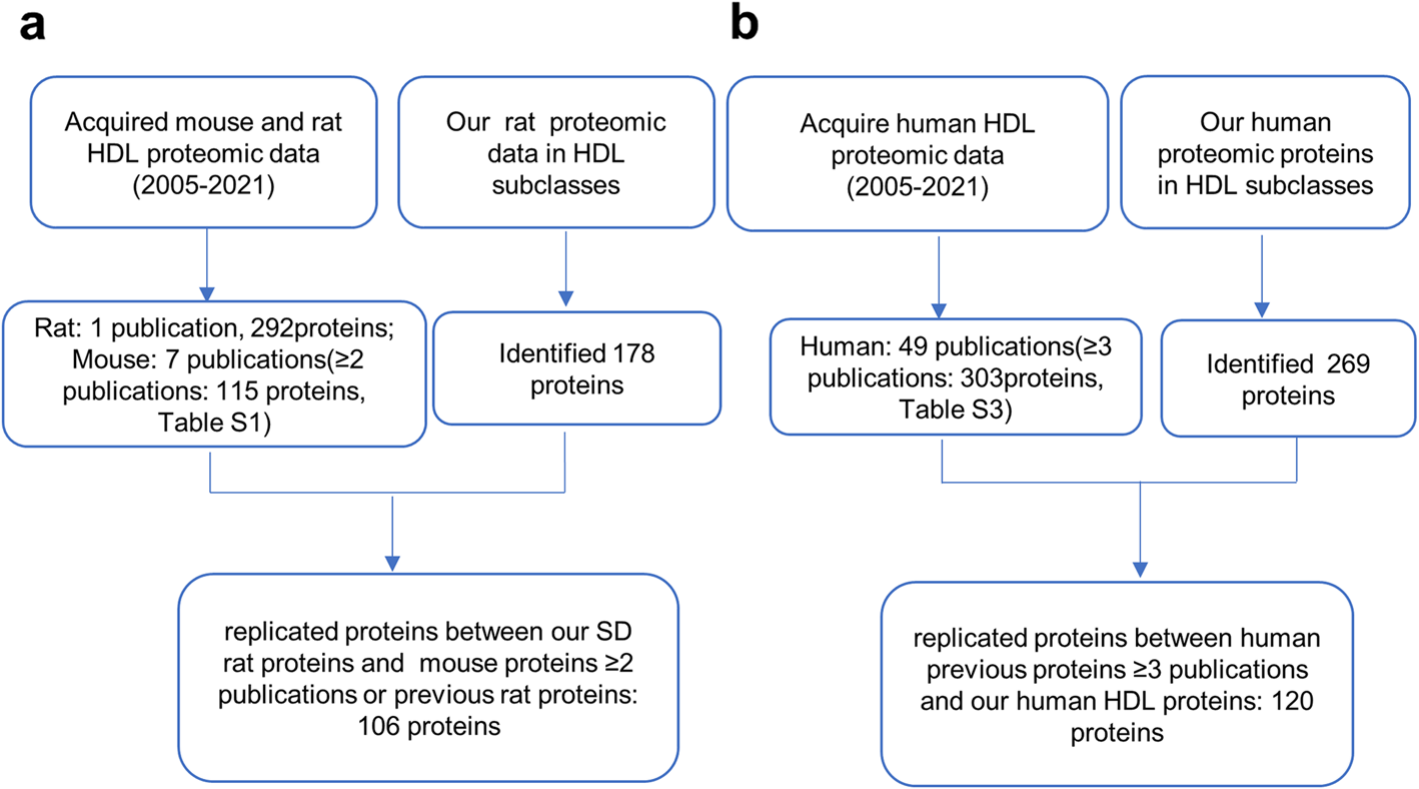
The strategies for identifying replicated HDL proteins. **a** The strategy for identifying replicated HDL proteins in rat samples. **b** The strategy for identifying replicated HDL proteins in human samples

By exploring previously published studies on HDL that used proteomics on human, mouse and rat samples (supplementary Tables S1-S4), the strategies for identifying replicated HDL proteins in human and rat samples are shown in Fig. 1.

### Identification of the relatively abundant proteins in the HDL subclasses

Due to a equal volume of the HDL subclasses was loading to MS detection, the MS proteomic content in different HDL subclasses could be interpreted as the concentration of the protein in different HDL subclasses. Therefore, the word “concentration” was used when explain the difference of the protein content in HDL subclasses. The changes in concentration of each particular protein between the HDL subclasses were analyzed by using “DEqMS” package [19] through R 4.1.2, and the relatively abundant proteins in each HDL subclass were defined as the proteins at higher concentration in that subclass than in the other two HDL subclasses. For example, the relatively abundant proteins of large HDL (L-HDL) identified in rat samples were the proteins that had higher concentration in the L-HDL subclass than in the M-HDL or S-HDL subclasses.

### Functional annotation of the relatively abundant proteins in each of the HDL subclasses

To distinguish the function of the relatively abundant proteins from each HDL subclass, the relatively abundant proteins were searched in Gene Ontology (GO) annotations website (https://www.ebi.ac.uk/QuickGO/annotations) for their biological processes. The GO terms that were enriched with these proteins fall within the following ten umbrella terms of known HDL-associated functions: lipid metabolism, blood coagulation, inflammation response, immune process, proteolysis regulation, complement system, antioxidation, apoptosis process, metal binding and vasodilation.

### Cholesterol efflux capacity (CEC) of the HDL subclasses

The HDL subclass-mediated CEC was detected according to previously study [20, 21]. Briefly, in a 48-well plate, 3 × 10^5^ J774 macrophages were plated and incubated with the radiolabeled high-glucose Dulbecco’s Modified Eagle’s Medium (DMEM) medium containing 10% fetal bovine serum (FBS), 2 µg/ml sterol O-acyltransferase 1 (ACAT) inhibitor (Sigma), 1 µCi/ml ^3^H-cholesterol (PerkinElmer, Boston, MA), and 0.3 mM 8-Br-cyclic adenosine monophosphate (cAMP) (Sigma) for 24 h. Followed by wash with phosphate-buffered saline (PBS) twice and synchronization with FBS-free medium for 4 h, the macrophages were treated with DMEM medium containing 0.5% bovine serum albumin (BSA) and 26.7% v/v of each HDL subclass for 4 h. Finally, the medium was gathered, while the macrophages were rinsed with PBS and then lysed with 1% Triton X-100. The radioactivity of the cell lysates and medium were respectively estimated on a liquid scintillation counter. CEC was expressed as ^3^H counts in efflux the medium relative to the total ^3^H counts in the cell lysates and medium together.

### The antioxidative capacity of each S/M/L-HDL subclass

HDL antioxidative capacity was quantified by measuring the capacity of HDL to mitigate the oxidation of oxidized low-density lipoprotein (ox-LDL) [22]. Ox-LDL (20 µg cholesterol) was added to each well. To measure the antioxidative capacity of the HDL subclasses, 20 µl of each S/M/L-HDL subclass was preincubated with ox-LDL for 1 h, followed by adding with 20 µl of dihydrorhodamine 123 (DHR, 50 µM) (Sigma) to be oxidized by ox-LDL. After incubation at 37 °C for 1 h, the oxidated DHR of each well was tested by fluorescence intensity on a SpectraMax M5 Reader (Molecular Devices) at an emission of 528 nm and an absorption of 485 nm. Finally, the antioxidative index was computed as follows: the endpoint fluorescence of the optical density of DHR incubated with ox-LDL divided by the optical density of DHR incubated with ox-LDL and each HDL subclass.

### Statistical analysis

The significantly changed proteins in concentration between the HDL subclasses were analyzed by R 4.1.0, and the cutoff values of log_2_FC and -log_10_(*P* value) were 1.5 and 3.0, respectively. Other experimental data are showed as the mean ± SEM. A two-tailed *P* < 0.05 was regarded to be significantly different in statistical analysis.

## Results

### Isolation and validation of the S/M/L-HDL subclasses in humans and rats

**Fig. 2:**
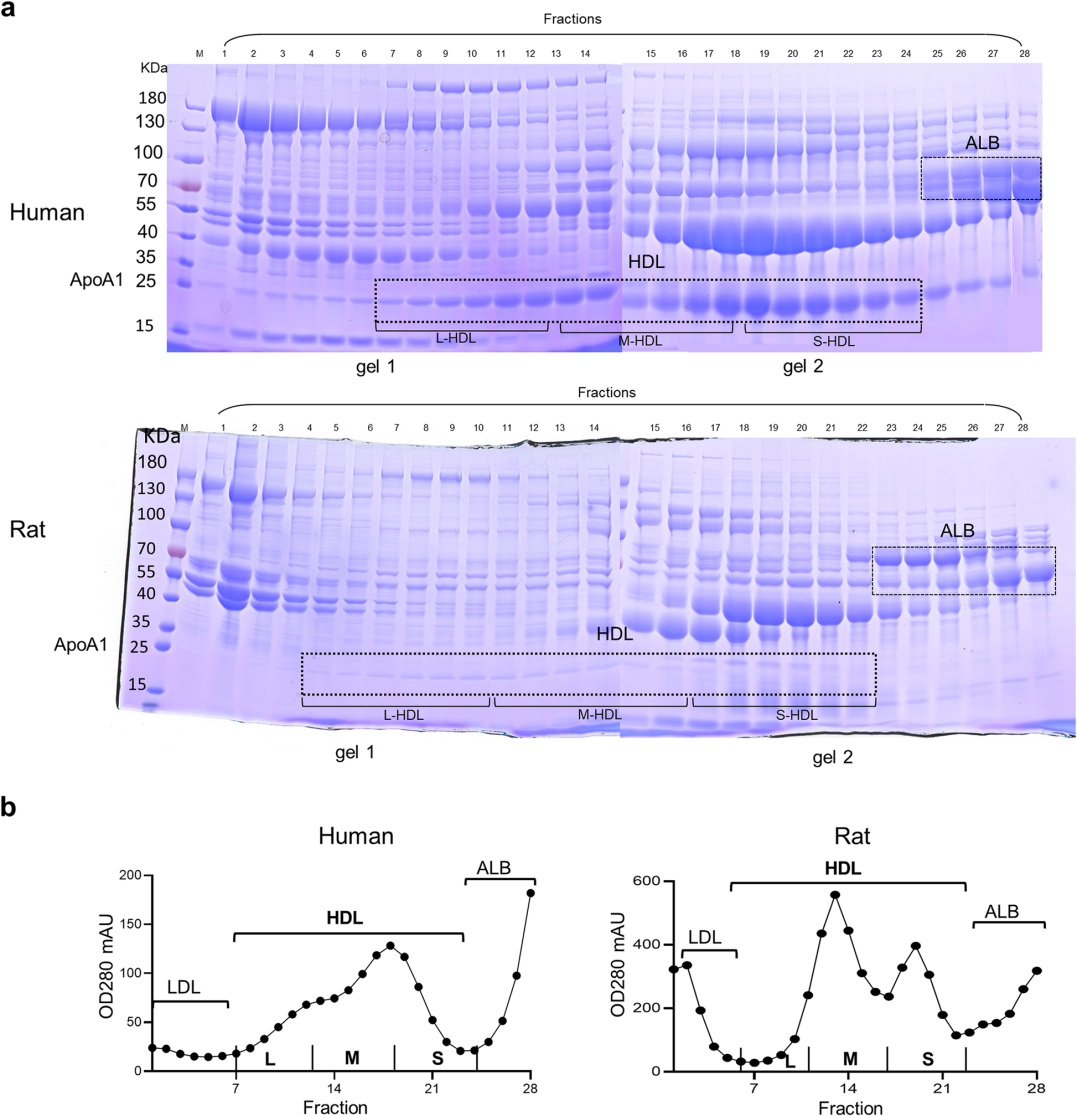
Validation of three Superdex 200 columns plus CSH purification for HDL subclasses. **a** SDS‒PAGE of the HDL fractions in human and rat samples. FPLC-isolated HDL subclasses were analyzed by SDS‒PAGE after incubation with CSH, which removed most of the non-phospholipid binding proteins. The gels were stained with Coomassie blue. Human HDL was in fractions 7–24 and was divided evenly into the L-HDL subclass (fractions 7–12), M-HDL subclass (fractions 13–18), and S-HDL subclass (fractions 19–24). The rat HDL was in fractions 5–22 and was divided evenly into L-HDL subclass (fractions 5–10), M-HDL subclass (fractions 11–16), and S-HDL subclass (fractions 17–22). **b** Protein contents of the HDL fractions in humans and rats. The FPLC-isolated HDL fractions were all obtained and their protein contents were measured by absorbing at OD280

The procedure using the three Superdex 200 column FPLC system with CSH was established to purify the HDL subclasses. Following collection, the fractions were dyed with Coomassie blue and a sodium dodecyl sulfate–polyacrylamide gel electrophoresis (SDS-PAGE) was run to detect specific HDL subclasses. As displayed in Fig. 2a, apolipoprotein (APO) A-I (APOA1) is the main protein of HDL, and its position is located at 25 kDa. Albumin (ALB) is located at 66 kDa, and the HDL fractions consistently contained APOA1 with little ALB. All HDL fractions were pooled into three subclasses evenly based on the size. As shown in Fig. 2a, Fractions 7–24 were classified as human HDL, in which fractions 7–12, fractions 13–18 and fractions 19–24 were collected as L-HDL, M-HDL and S-HDL, respectively. Whereas, fractions 5–22 were classified as rat HDL, in which fractions 5–10, fractions 11–16 and fractions 17–22 were collected as L-HDL, M-HDL, and S-HDL, respectively. When protein contents of the collected HDL fractions from human and rat samples were analyzed, there were two distinct peaks (Fig. 2b); however, the peak between S-HDL and M -HDL subclasses was higher than that between M-HDL and L-HDL subclasses in human, while the peak between M-HDL and L-HDL subclasses was higher than that between S-HDL and M-HDL in rat, which indicated that the protein concentrations in the three HDL subclasses from human and rat samples were inconsistent.

### Identified proteins of the S/M/L-HDL subclasses in human and rat samples

**Fig. 3:**
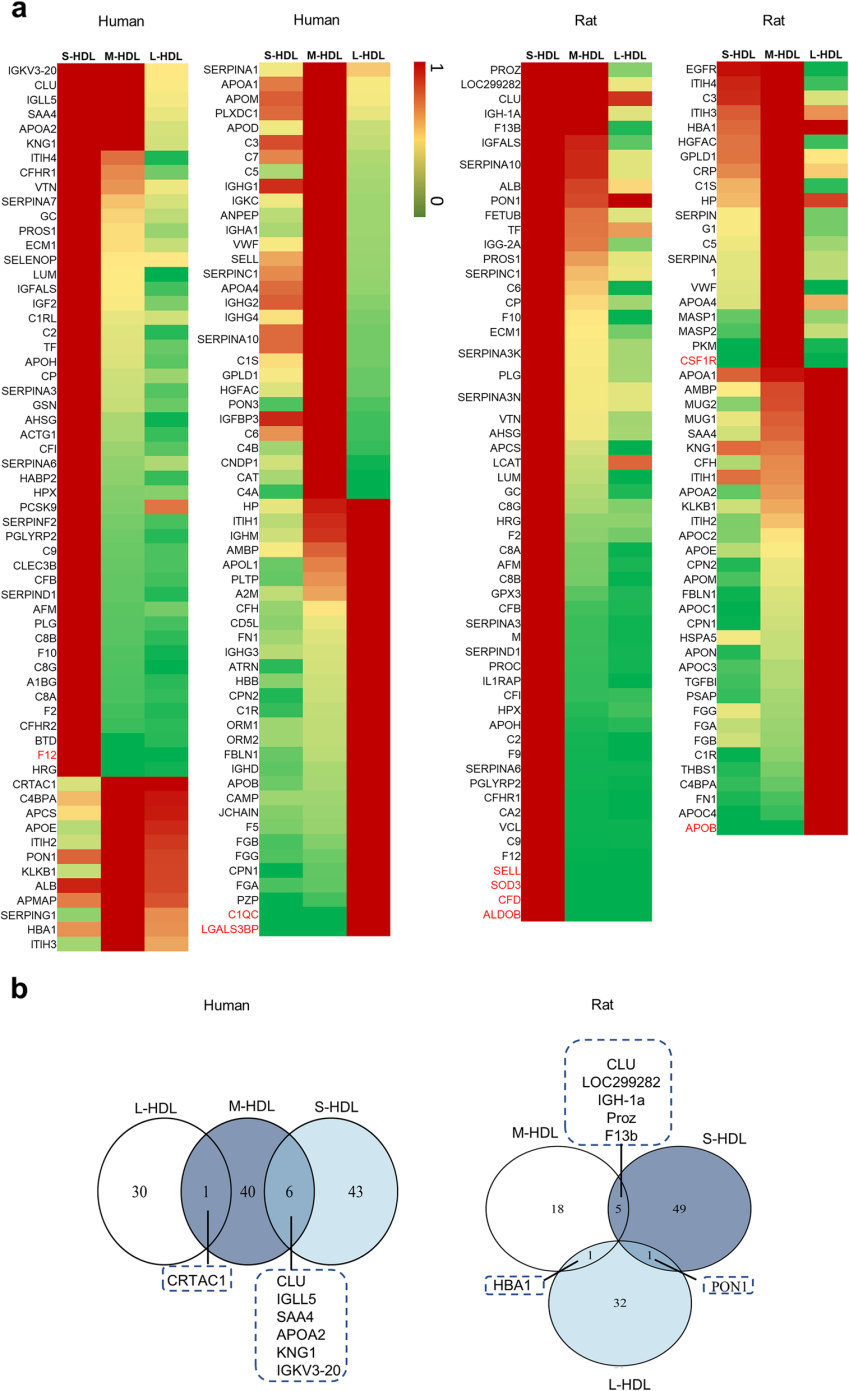
The identified proteins of the S/M/L-HDL subclasses in human and rat samples. **a** Heatmap of the ratios of the sum of peptide count for each protein in the S/M/L-HDL subclasses from human and rat samples. A equal volume of HDL subclasses were applied to the CSH resin, trypsinized, and then identified via LC‒MS/MS. The ratios of the sum of peptide count for each protein in the three HDL subclasses were calculated according to the unique peptide count of MS data, and a value of 1.0 was assigned to the subclass containing the highest peptide count for that particular protein and the peptide count of the other subclasses were scaled accordingly. the highest ratio (1.0) are colored red and gradually changed to yellow for the lower values, the lowest ratio (0.0) are colored green. The distinct proteins in the HDL subclass are shown in red font. **b** The distribution and overlaps of the proteins with the peptide count ratio of 1.0 among the HDL subclasses. The venn diagrams of the proteins with the peptide count ratio of 1.0 in each S/M/L-HDL subclass were drawn

The HDL proteins from human and rat were defined by searching previously published human, mouse and rat HDL proteomic studies (Supplemental Tables S1-S4) for replicate proteins. According to the strategy shown in Fig. 1, it was found that there were 120 and 106 proteins in the HDL subclasses in human and rat samples, respectively. The heatmap of the ratios of the protein peptide count in the three HDL subclasses was drawn according to unique peptide counts (Table S5-S6), and a value of 1.0 was assigned to the subclass containing the highest peptide count for the particular protein and the peptide counts of the other two subclasses were scaled accordingly. As shown in Fig. 3a. Forty-nine proteins, 47 proteins, and 31 proteins had the peptide count ratio of 1.0 in the human S-HDL, M-HDL, and L-HDL, respectively; moreover, 6 proteins (immunoglobulin lambda-like polypeptide 5 (IGLL5), clusterin (CLU), serum amyloid A4 (SAA4), apolipoprotein A2 (APOA2), Kininogen 1(KNG1), immunoglobulin kappa variable 3–20 (IGKV3-20)) had the peptide count ratio of 1.0 overlapped in the M-HDL and S-HDL subclasses, and 1 protein (cartilage acidic protein 1 (CRTAC1)) had the peptide count ratio of 1.0 overlapped in the M-HDL and L-HDL subclasses. However, there was no protein with the peptide count ratio of 1.0 overlapped in the L-HDL and S-HDL subclasses (Fig. 3b). In rats, there were 55 proteins, 24 proteins, and 34 proteins that had the peptide count ratio of 1.0 in the S-HDL, M-HDL, and L-HDL subclasses, respectively. Five proteins (vitamin K-dependent protein Z (Proz), CLU, serine protease inhibitor (LOC299282), immunoglobulin gamma-2B chain C region (IGH-1a), and coagulation factor XIII B chain (F13b)) with the peptide count ratio of 1.0 overlapped in the M-HDL and S-HDL subclasses, 1 protein (hemoglobin subunit alpha-1 (HBA1)) with the peptide count ratio of 1.0 overlapped in the M-HDL and L-HDL subclasses, and 1 protein (PON1) with the peptide count ratio of 1.0 overlapped in the L-HDL and S-HDL subclasses (Fig. 3b). These data indicated that the proteomics of the S/M/L-HDL subclasses from human and rat samples was different, with more proteins with the peptide count ratio of 1.0 were distributed in the S/M-HDL subclasses than in the L-HDL subclass in human, while more proteins with the peptide count ratio of 1.0 were distributed in the S/L-HDL subclasses than in the M-HDL subclass in rat.

#### The changes in concentrations of each particular protein in S/M/L-HDL subclasses in human and rat samples

**Fig. 4:**
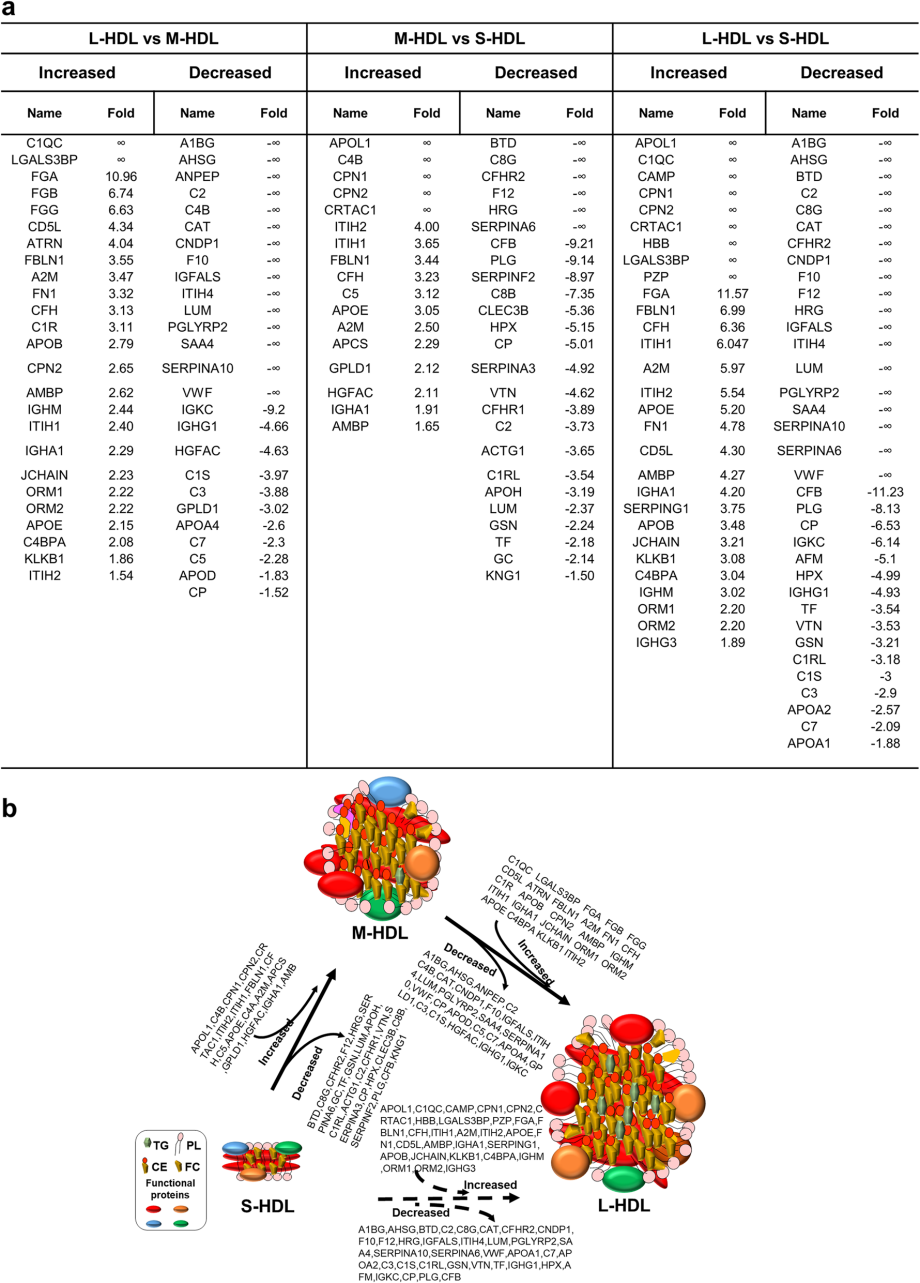
The significantly changed proteins and dynamic protein distribution in the S/M/L-HDL subclasses from humans. **a** The significantly changed proteins in concentration between the HDL subclasses. The comparison of protein contents were analyzed by R, and the cutoff values of log_2_FC and -log_10_(*P* value) were 1.5 and 3.0, respectively. Log_2_ FC represents the logarithm value of fold change of the proteins that significantly changed in concentration between the S/M/L-HDL subclasses. **b** The dynamic distribution of the significantly changed proteins during HDL maturation in human samples. The dynamic distribution of the S-HDL to M-HDL subclasses and the M-HDL to L-HDL subclasses are indicated by the solid arrow, and the dynamic distribution of the S-HDL to L-HDL subclasses is indicated by the dotted arrow

**Fig. 5:**
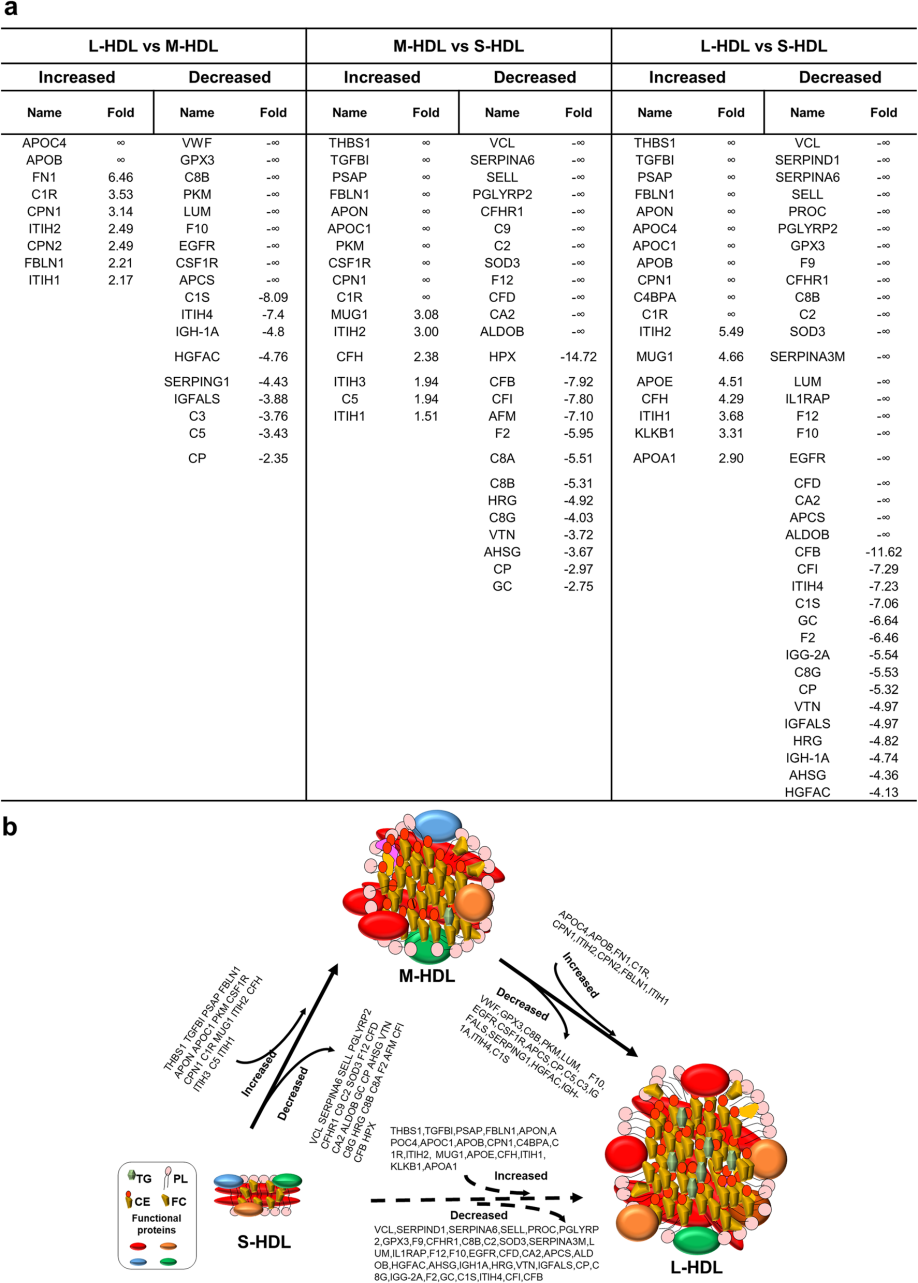
The significantly changed proteins and dynamic protein distribution in the S/M/L-HDL subclass from rats. **a** The significantly changed proteins in concentration between the HDL subclasses. The comparisons of protein contents were analyzed by R, and the cutoff values of log_2_FC and -log_10_(*P* value) were 1.5 and 3.0, respectively. Log_2_ FC represents the logarithm value of fold change for the proteins that significantly changed in concentration between the S/M/L-HDL subclasses. **b** The dynamic distribution of the significantly changed proteins in concentration during HDL maturation in rats samples. The dynamic distribution of the S-HDL to M-HDL subclasses and the M-HDL to L-HDL subclasses are indicated by the solid arrow, and the dynamic distribution of the S-HDL to L-HDL subclasses is indicated by the dotted arrow

Results from the proteomic comparison of the HDL subclasses in human samples is shown in Fig. 4a. Compared with the human M-HDL subclass, 25 proteins were increased and 26 proteins were decreased in the human L-HDL subclass. Compared with the human S-HDL subclass, 17 proteins were increased and 25 proteins were decreased in the human M-HDL subclass, while 29 proteins were increased and 35 proteins were reduced in the L-HDL subclass. Figure 4b models the dynamic distribution of the changed proteins in concentration between the three subclasses in human samples. For the proteomic comparison of the HDL subclasses in rat samples (Fig. 5a), compared with the rat M-HDL subclass, 9 proteins were increased and 18 proteins were decreased in the rat L-HDL subclass, and compared with the rat S-HDL subclass, 16 proteins were increased and 25 proteins were decreased in the rat M-HDL subclass, while 18 proteins were increased and 37 proteins were decreased in the rat S-HDL subclass. Meanwhile, a model representing the dynamic distribution of changed proteins in concentration between the three subclasses during HDL maturation in the rats was established (Fig. 5b). These results revealed that the amount of the changed proteins in content between the L-HDL vs. S-HDL group was greater than that in the other two groups (L-HDL vs. M-HDL, M-HDL vs. S-HDL), either in human or rat samples.

#### The relatively abundant proteins of each S/M/L HDL subclass in human and SD rat samples

The relatively abundant proteins were defined as those proteins that were at higher concentration in one HDL subclass than in the other two HDL subclasses based on the results shown in Fig. 4a and Fig. 5a. For example, the relatively abundant proteins in the rat L-HDL subclass included the proteins that has higher concentration in the L-HDL subclass than in the M-HDL or the S-HDL subclasses. The relatively abundant proteins in each HDL subclass are shown in Table 1.

**Table 1:** The relatively abundant proteins in each HDL subclass (vs. the other two subclasses) in human and rats samples

**Species**	**L-HDL**	**M-HDL**	**S-HDL**	**Total**
Human	C1QC, GLIPR2, LGALS3BP,THBS1, TLN1, TTR, FGA, FGB, FGG, CD5L, ATRN, FBLN1, A2M, FN1, CFH, C1R, APOB, CPN2, AMBP, IGHM, ITIH1, IGHA1, JCHAIN, ORM1, ORM2, APOE, C4BPA, KLKB1, ITIH2, APOL1, APOL1, CAMP, CPN1, CRTAC1, PZP, SERPING1, IGHG3, HBB, HLA-A	A1BG, AHSG, ANPEP, C2, C4B, CAT, CNDP1, F10, IGFALS, ITIH4, LUM, PGLYRP2, SAA4, SERPINA10, VWF, APOL1, CPN1, CPN2, CRTAC1, CP, APOD, C7, APOA4, GPLD1, C3, C1S, HGFAC, IGHG1, IGKC, ITIH2, ITIH1, FBLN1, CFH, C5, APOE,C4A, A2M, APCS, IGHA1, AMBP	BTD, C8G, CFHR2, F12, HRG, SERPINA6, VCL, A1BG, AHSG, C2, CAT, CNDP1, F10, IGFALS, ITIH4, LUM, PGLYRP2, SAA4, SERPINA10, VWF, GC, KNG1, APOH, C1RL, ACTG1, CFHR1, VTN, SERPINA3, HPX, CLEC3B, C8B, SERPINF2, PLG, APOA1, C7, APOA2, C3, C1S, GSN, TF, IGHG1, AFM, IGKC, CP, CFB	85
Rat	APOC4, APOB, THBS1, TGFBI, PSAP, FBLN1, APON, APOC1, CPN1, C4BPA, C1R, FN1, ITIH2, MUG1, APOE, CFH, ITIH1, KLKB1, APOA1, CPN2	THBS1, TGFBI, PSAP, FBLN1, APON, APOC1, PKM, CSF1R, CPN1, C1R, VWF, GPX3, C8B, LUM, F10, EGFR, APCS, C1S, ITIH4, IGH-1A, HGFAC, SERPING1, IGFALS, C3, C5, MUG1, ITIH2, CFH, CP, ITIH3, ITIH1	VCL, SERPINA6, SELL, PGLYRP2, CFHR1, C9, C2, SOD3,F12, CFD, CA2, ALDOB, SERPIND1, PROC, GPX3, F9, C8B, SERPINA3M, LUM, IL1RAP, F10, EGFR, APCS, HPX, CFB, CFI, ITIH4, AFM, C1S, GC, F2, IGG-2A, C8G,C8A, CP, IGFALS, VTN, HRG, IGH-1A, AHSG, HGFAC	68

As described in Figs. 4a and 5a, 85 proteins and 68 proteins were identified to be significantly changed in concentration in the HDL subclasses from human and rat samples, respectively. The relatively abundant proteins in each HDL subclass were defined as the proteins at higher concentration in that subclass than in the other two HDL subclasses. For example, the relatively abundant proteins of L-HDL identified in rat samples were the proteins that has higher concentration in the L-HDL subclass than in the M-HDL or S-HDL subclasses

**Fig. 6:**
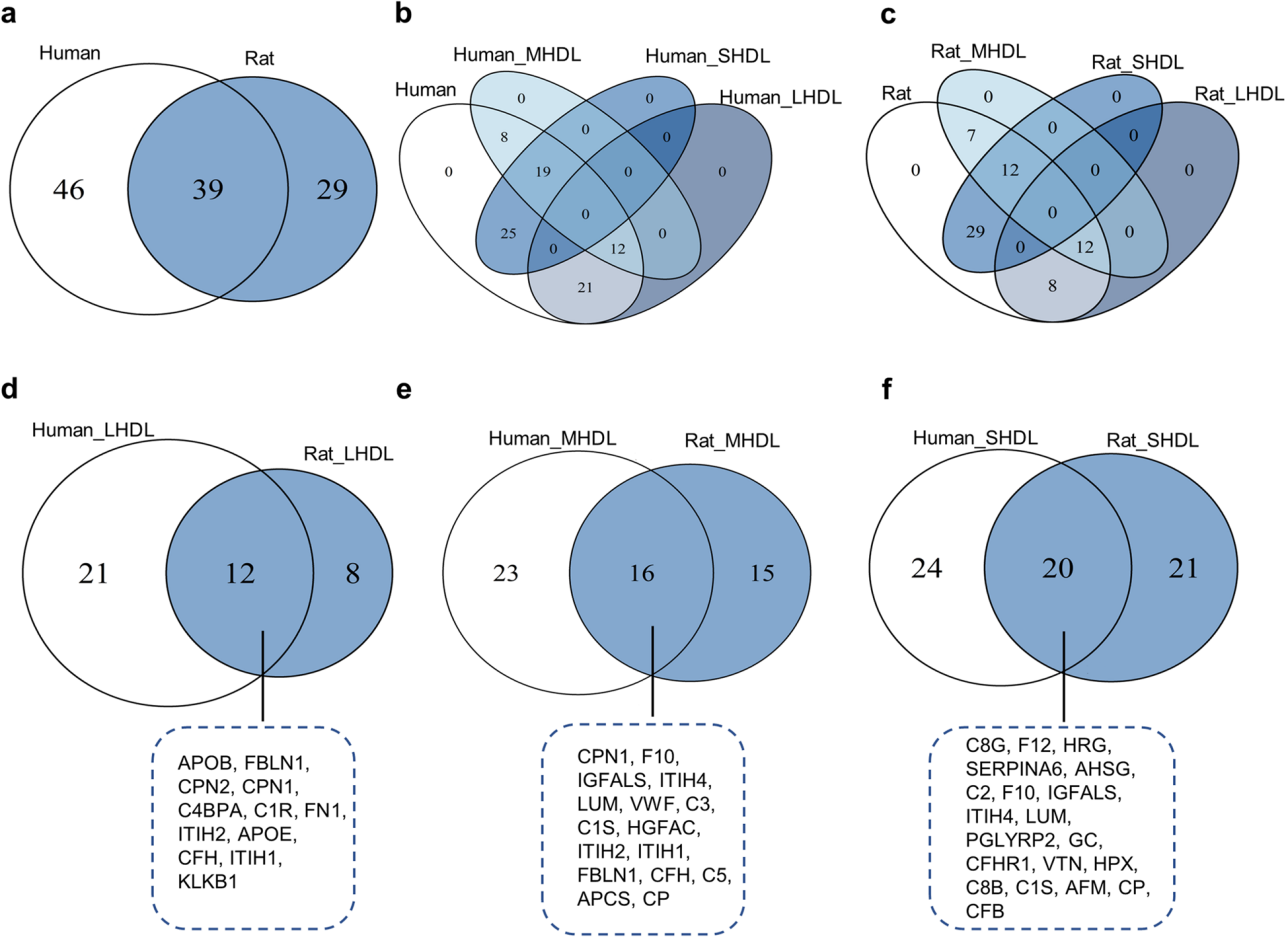
Overlapping and distinct proteins of the relatively abundant proteins in each S/M/L-HDL subclass from human and rats. **a** Venn diagram of the significantly changed proteins in concentration in the HDL subclasses of human and rat samples. **b** Venn diagram of the relatively abundant proteins in each human S/M/L-HDL subclass. **c** Venn diagram of the relatively abundant proteins in each rat S/M/L-HDL subclass. **d** Venn diagram of the relatively abundant proteins in the L-HDL subclass in human and rat samples. **e** Venn diagram of the relatively abundant proteins in the M-HDL subclass in human and rat samples. **f** Venn diagram of the relatively abundant proteins in the S-HDL subclass in human and rat samples

The relatively abundant proteins in each of the HDL subclasses was compared between human and rat samples, as shown in Fig. 6a. In total, 39 proteins changed in concentration in the HDL subclasses overlapped in human and rat samples, and 46 proteins were distinctly and changed in concentration among human HDL subclasses, whereas 29 proteins were distinctly changed in concentration among rat HDL subclasses. A Venn diagram of the relatively abundant proteins of each subclass in human samples (Fig. 6b) showed that there were 12 relatively abundant proteins overlapping in the M-HDL and L-HDL subclasses and 19 relatively abundant proteins overlapping in the M-HDL and S-HDL subclasses. None of the relatively abundant proteins overlapped in the human L-HDL and S-HDL subclasses. A Venn diagram of the relatively abundant proteins of each HDL subclass in rat samples (Fig. 6c) showed that there were 12 relatively abundant proteins overlapping in the L-HDL and M-HDL subclasses, as well as in the M-HDL and S-HDL subclasses. None of the relatively abundant proteins overlapped in the rat L-HDL and S-HDL subclasses. The results indicated that more than 10 relatively abundant proteins overlapped in the S-HDL and M-HDL subclasses, as well as in the L-HDL and M-HDL subclasses; however, none of the relatively abundant proteins overlapped in the L-HDL and S-HDL subclasses in either human or rat samples. These data suggest that the L-HDL and S-HDL subclasses are likely to have different proteomic components during HDL maturation in both humans and rats.

Additionally, the relatively abundant proteins in the HDL subclasses in human and rat samples that were distinct or overlapping was also displayed using a Venn diagram. There were 12 relatively abundant proteins in the L-HDL subclass that overlapped in the samples of human and rat (Fig. 6d), 16 relatively abundant proteins in the M-HDL subclass that overlapped in the samples of human and rat (Fig. 6e), and 20 relatively abundant proteins in the S-HDL subclass that overlapped in the samples of human and rat (Fig. 6f).

### Functional annotation of the relatively abundant proteins in each HDL subclass in human and rat samples

The relatively abundant proteins in each HDL subclass were searched for GO to distinguish the biological function. The GO terms enriched with the identified proteins fall within the following ten umbrella terms of known HDL-associated functions: lipid metabolism, blood coagulation, inflammation response, immune process, proteolysis regulation, complement system, antioxidation, apoptosis process, metal binding and vasodilation.

**Fig. 7:**
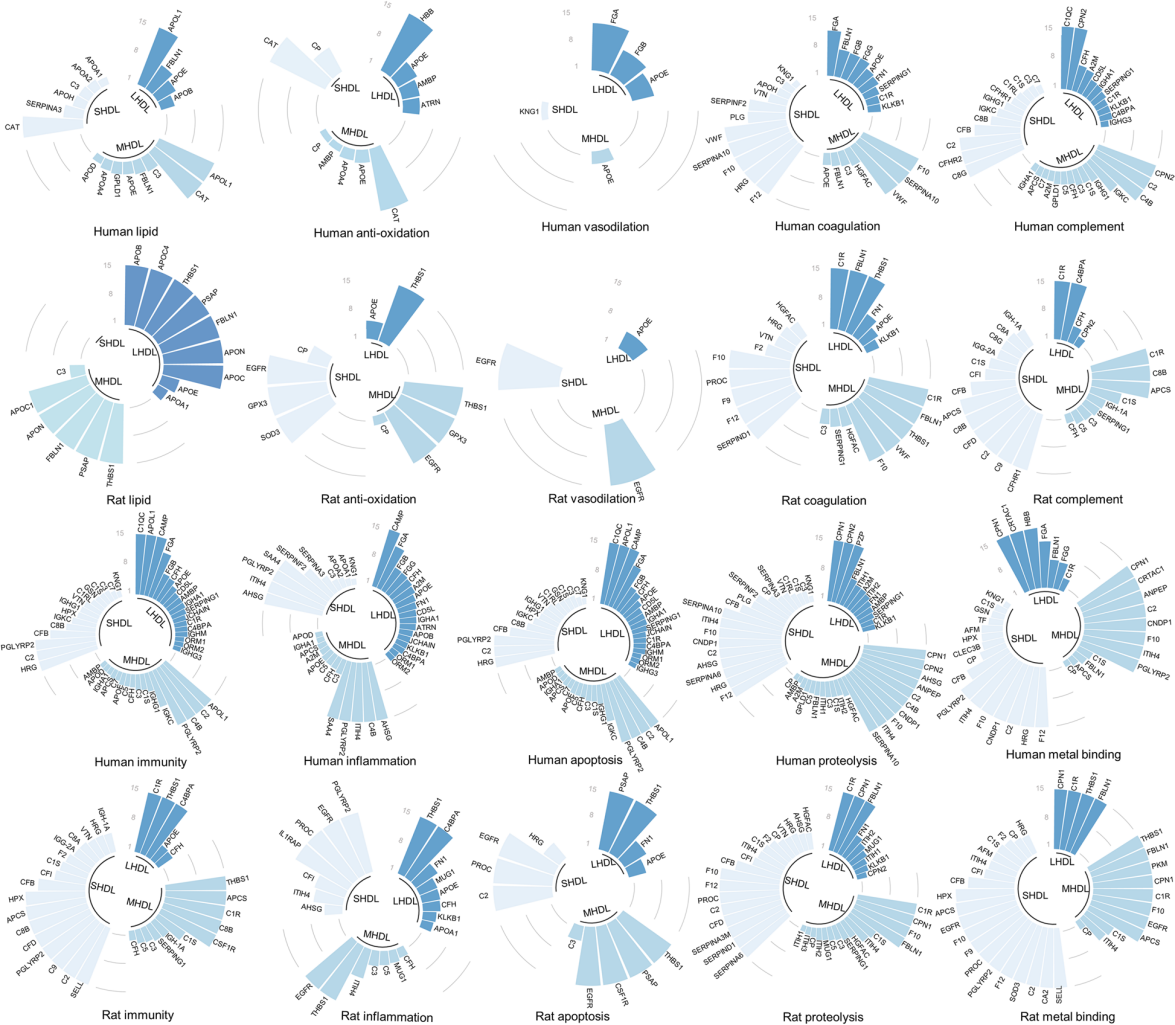
Functional annotation of the relatively abundant proteins in each HDL subclass in human and rats. The relatively abundant proteins in the S/M/L-HDL subclasses, as shown in Table 1, were searched in Gene Ontology (GO) annotations website (https://www.ebi.ac.uk/QuickGO/annotations), and the GO-molecular function and GO-biological process were grouped under ten umbrella terms. The relatively abundant proteins of the HDL subclasses are represented by different colored bars, and if none of the relatively abundant proteins in the HDL subclass grouped in GO terms, the proteins were represented by blank bars. The length of the bar represents the enriched log_2_FC value of the protein in each HDL subclass, and the critical maximum of the log_2_FC value (value 15) is marked as ∞

As shown in Fig. 7, the relatively abundant proteins of the S/M/L-HDL subclasses, including 68 proteins in rat samples and 85 proteins in human samples (Table 1), were involved in the ten HDL-related biological functions. In terms of the complement, inflammation, proteolysis regulation, coagulation, immune process and apoptotic process biological functions, the amount of the relatively abundant proteins in the three HDL subclasses participating in these biological processes was not obviously different, either in human or rat samples. However, it was revealed that the relatively abundant proteins of the M/L-HDL subclasses played primary roles in the function of vasodilation, but not those in S-HDL subclass in human. Meanwhile, the number of the involved proteins distributed among the S/M/L-HDL subclasses in rat samples was not obviously different. Regarding HDL antioxidation, the number of proteins involved that belonged to the M/L-HDL subclass was greater than those that belonged to the S-HDL subclass in human samples, whereas the number of antioxidative proteins in the L-HDL subclass was the lowest among the S/M/L-HDL subclasses in rat samples. Moreover, there were more relatively abundant proteins participating in the process of metal binding in the S/M-HDL subclasses than that in the L-HDL subclass, in both human and rat samples, and there were more relatively abundant proteins participating in lipid metabolism distributed in the M/L-HDL subclasses than in the S-HDL subclass in rat samples. It was concluded that the relatively abundant proteins exerting the biological function of vasodilation and antioxidation were enriched in the M/L-HDL subclasses, and barely found in the S-HDL subclass in human samples. However, in rat samples, antioxidative proteins were more enriched in the M-HDL subclass but less enriched in the L-HDL subclass, while lipid metabolism-associated proteins were more enriched in the M/L-HDL subclass but less enriched in the S-HDL subclass.

#### CEC of the HDL subclasses in human and rat samples

**Fig. 8:**
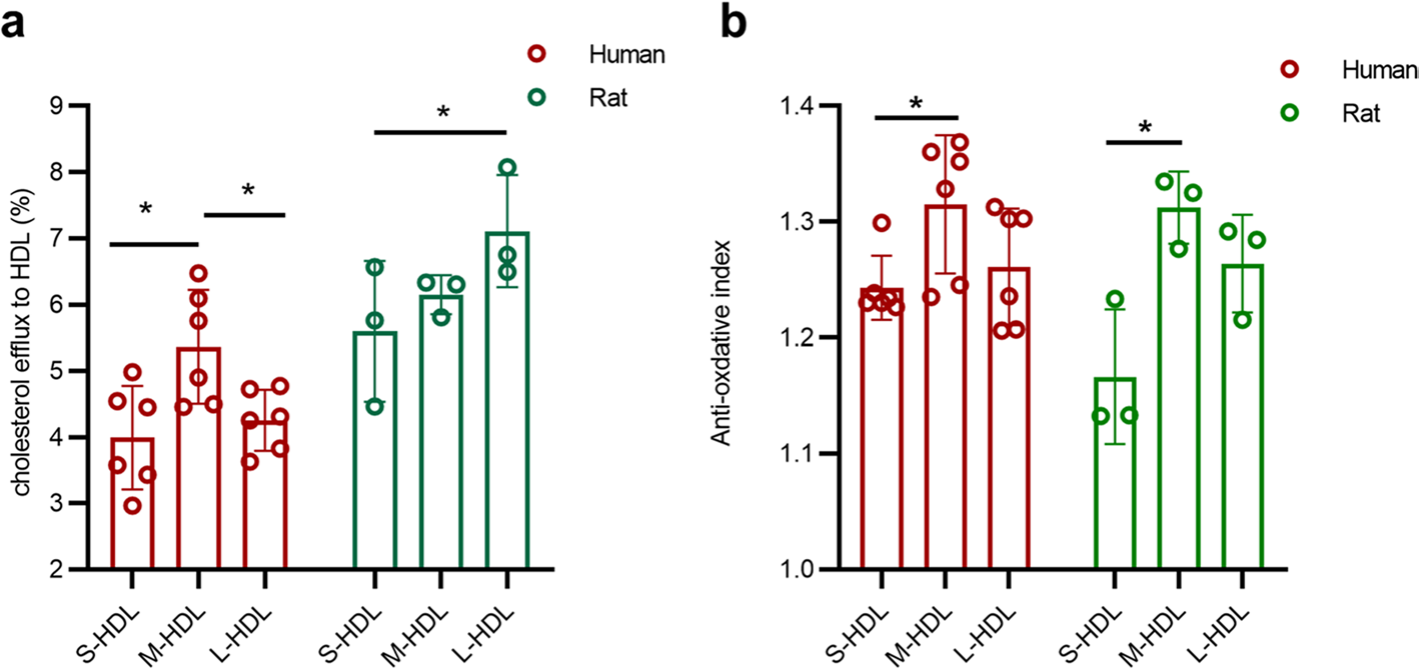
Cholesterol efflux and antioxidative capacities of each HDL subclass in human and rat samples. **a** Cholesterol efflux capacity of the S/M/L-HDL subclasses in human and rat samples. HDL subclasses were separated by FPLC and prepared for cholesterol efflux examination. J774A.1 cells were preloaded with ^3^H-cholesterol (1 Ci/ml) for 24 h. ^3^H-cholesterol efflux was detected after incubation with a equal volume of HDL subclass for 4 h. **b** Antioxidative capacity of the S/M/L-HDL subclasses in human and rat samples. HDL antioxidative ability was reflected as the antioxidative index, which was calculated as the endpoint fluorescence of DHR incubated with ox-LDL corrected for the optical density and divided by the optical density of DHR coincubated with ox-LDL and a equal volume of HDL subclass. *, *P* < 0.05

The CEC of the S/M/L-HDL subclasses in human and rat samples were detected by measuring the ^3^H cholesterol efflux from J77 4.1 macrophages. As shown in Fig. 8a, the M-HDL subclass has the highest CEC among the S/M/L-HDL subclasses in human samples, whereas the L-HDL subclass has a higher CEC than the S/M-HDL subclasses in rat samples. Notably, this trend was similar to that of the APOA1 content in the heatmap of the S/M/L-HDL subclasses, both in human and rat samples.

#### Antioxidative index of HDL subclasses in human and rat samples

HDL antioxidative ability was reflected as the antioxidative index. It was found that the M-HDL subclass had a higher antioxidative capacity than the S-HDL subclass in both human and rat samples (Fig. 8b), which was consistent with the finding that the relatively abundant proteins grouped in antioxidation function in the S/M/L-HDL subclasses in human and rat samples.

## Discussion

Recently, many researchers [12, 23–27] have become interested in investigating the role of HDL subclasses and their proteomic alterations played in the HDL atherogenic and non-atherogenic properties. Based on the previous procedures that were developed to separate mouse HDL subclasses and characterize the proteomic components of the HDL subclasses, the differences of proteomics and functions between the HDL subclasses in human and rat samples were explored in the present study. The MS results identified 120 and 106 proteins in the HDL subclasses, and 85 and 68 proteins were changed in concentration between the S/M/L-HDL subclasses in human and SD rat samples, respectively. Interestingly, it was found that the relatively abundant proteins in the S-HDL subclass and the L-HDL subclass were totally different, in both human and rat samples. The searched biological functions of the relatively abundant proteins revealed that the relatively abundant proteins participated in the biological functions of vasodilation and antioxidation were enriched in the M/L-HDL subclasses, and rarely found in the S-HDL subclass, while lipid metabolism enriched in S/M-HDL, in human samples. However, in rat samples, antioxidative proteins were more enriched in the M/S-HDL subclasses but less enriched in the L-HDL subclass, while the proteins involved in lipid metabolism were more enriched in the M/L-HDL subclasses but little enriched in the S-HDL subclass. Among the three HDL subclasses, it was proved that the M-HDL and L-HDL possessed the highest CEC in human and rat samples, respectively, while the M-HDL subclass exhibited a relatively higher antioxidative capacity both in human and rat samples, which were consistent with the lipid metabolism and antioxidative function of the relatively abundant proteins distributed among the three HDL subclasses.

Not only can different diseases [28, 29] cause specific proteomic changes in HDL, but these changes can also vary in the same disease. In studies of heart failure, Andreas Oberbach [30] found that the levels of several proteins (including alpha-2-Macroglobulin (A2M), integrin alpha-2 (ITGA2), and amyloid-beta A4 precursor protein-binding family A member 1 (APBA1)) varied, as well as bacterial proteins were detected in HDL in chronic heart failure patients. Emmens, J. E [31]. discovered that one of the strongest contributors to heart failure was the expressions of pulmonary surfactant-associated protein B and filamin-A in HDL. In the area of chronic kidney disease, Shao, B. [32] reported increased levels of β2-microglobulin (B2M), alpha-1-microglobulin/bikunin precursor (AMBP), cystatin-C (CST3), retinol-binding protein 4 (RBP4), and complement factor D (CFD) in HDL particles from chronic renal failure patients, while Mangé et al. [33] found 21 decreased and 19 increased proteins in concentration in HDL particles from hemodialysis patients. However, the finding in Nans Florens’s study [34] that found that among the proteins identified by Mangé et al., only two proteins (AMBP and B2M) were found to be significantly changed, is controversial. Moreover, Baohai Shao et al. [24] discovered an association between the levels of HDL-associated proteins (including PON1, paraoxonase-3 (PON3), and lecithin cholesterol acyl transferase (LCAT)) and cardiovascular risk in chronic kidney disease patients. This evidence suggests that HDL proteomic changes differ in a variety of disease statuses, and it is proposed that the diversity is not only due to the differences in diseases, but also due to the purification methods used for HDL or HDL subclasses.

There are several methods used for HDL isolation, including the traditional HDL isolation methods of density gradient ultracentrifugation (UC) and size-exclusion chromatography (SEC) or FPLC separation. The study by Riwanto [35] showed that the concentrations of apolipoprotein C- III (APOC3) and CLU identified in HDL when isolated by UC and FPLC are similar, but other studies [18, 36] have shown that when HDL is isolated by SEC or FPLC, there are unique proteins identified compared to when UC is used for isolation, and certain proteins may not overlap with the proteins identified using traditional HDL isolation methods. For example, the recent research [17] by Yong-Hyun Han identified 289 proteins of UC-purified HDLs, and 321 proteins of FPLC-purified HDL subclasses in human samples; moreover, lipopolysaccharide-binding protein (LBP) was particularly enriched in FPLC-purified HDL3, but notably absent in UC-purified HDLs. Therefore, since HDL is greatly heterogeneous in their composition and size, it is more reasonable to conduct proteomic study of HDL subclasses based on FPLC-purified method.

Many studies have shown that HDL-related proteins are distributed in distinct patterns across their sizes; however, the data even with using specific HDL purification methods are inconsistent. In early exploration of the protein content in the UC-isolated HDL particles, the MS data reveal that HDL3 is significantly enriched with proteins related to lipid metabolism/transport [37, 38] and antioxidation [37, 39], while HDL2 is enriched with APOC, apolipoprotein E (APOE), SAA, and apolipoprotein (a) (LPA) [37, 38, 40]. However, the reports on apolipoprotein A-II (APOA2) and apolipoprotein D (APOD) enrichment in HDL2 or HDL3 have been inconsistent [37, 40]. The proteomic alterations between the HDL subclasses when purified by FPLC or SEC seem to be more complicated than when purified by UC due to the identification of more HDL proteins. A study [23] of HDL fractions purified by SEC detected 150 proteins in humans, and the study [18] that first purified HDL by three superdex 200 FPLC identified 47 proteins in HDL subclasses is largely in accordance with the present findings. A recent study [17] using both the FPLC and UC methods for purification verified that PON1, SERPINA1, and phospholipid transfer protein (PLTP) are enriched in HDL3, which is not completely similar to the present human HDL heatmap. PON1 and SERPINA1 seemed to be enriched more in the M-HDL subclass, while PLTP was enriched in the L-HDL subclass, although the three proteins were not significantly changed in concentration between the S/M/L-HDL subclasses. These differences may be due to the different FPLC separation methods of the HDL subclasses, either with a gel filtration column [17] or three Superdex 200 FPLC columns (used in the present study), and the different classifications of HDL subclasses (HDL2/3 vs. S/M/L-HDL). Besides, there was only one rat proteomics study [15] on HDL but not on HDL subclasses, the present findings displayed that it was inconsistent in lipid metabolism between rat and human samples, and the detailed mechanism needs further investigation, for example, while APOA1 enriched most in the M/S-HDL subclasses and less in the L-HDL subclass in human samples, similarly to other human and mouse studies [16, 17, 23], APOA1 was more highly enriched in the L-HDL subclass than in the S-HDL subclass in rat samples. However, the present data interestingly indicated in the S-HDL subclass and the L-HDL subclass were profoundly different based on the relatively abundant proteins of each HDL subclasses, in both human and rat samples, which happened to coincide with the physiological process of HDL maturation, and in turn suggested this purified method was reasonable and came in line with reality.

Discovering the proteomic changes in the HDL subclasses seems very effective for evaluating the function of the HDL subclasses; for example, Gordon, S. M. [41] discovered that rosuvastatin treatment resulted in increase of SERPINA1 in SEC-isolated L-HDL particles and enhanced HDL anti-inflammatory functionality, and Dr. Heinecke [11] found that diabetes impaired cholesterol efflux to S-HDL particles through the alteration of APOC2 and SERPINA1 levels in S-HDL. Dr. Vaisar Tomas [12] discovered that elevated concentrations of M-HDL subclass, as well as the abundance PON1 in HDL could protect diabetes patients from vascular complications (independent of HDL-C). Therefore, it will be of great importance to evaluate the specific function of the HDL subclasses by determining the relative abundance of associated proteins among the HDL subclasses.

This study presented that the relatively abundant proteins participated in lipid metabolism through searching for GO annotation enriched in M/S-HDL and M/L-HDL subclasses in human and rat samples, respectively. Furthermore, the M-HDL and L-HDL were proved to exhibit relatively higher CEC among the three HDL subclasses in humans and rats, respectively, which was similar to the trend of APOA1 contents in S/M/L-HDL in the heatmap and was in line with other study [42]. However, the proteomic compositions of the HDL subclasses related to CEC are inconsistent, in both patients with healthy and diseased status. An HDL proteomic study of healthy humans [42] found that APOA1, APOA2, immunoglobulins, and serum amyloid P (SAP) are strongly correlated with CEC, while Scott M. Gordon [43] discovers that the there are 10 HDL proteins (APOA1, APOC3, APOA2, apolipoprotein C -I (APOC1), antithrombin-III (ANT3), PON1, alpha-2-antiplasmin (A2AP), RBP4, apolipoprotein A -IV (APOA4), and macrophage-capping protein (GELS)) and 6 proteins (immunoglobulin heavy constant gamma 1( IGHG1), complement C-4A, complement C2, complement C9, Ceruloplasmin (Cp), and kallikrein) associated with increased CEC and decreased CEC, respectively. Tan, Y et al. [44] discovered that the CEC of HDL2 and HDL3 isolated by UC are similar both in acute coronary syndrome (ACS) patients and healthy controls; however, the CEC of both HDL3 and HDL2 from the patients with ACS are significantly lower than those from healthy controls, and it is detected in ACS subjects that 9 proteins (fibrinogen gamma chain(Fgg), alpha-1B-glycoprotein, APOA1, APOA4, APOE, apolipoprotein L1 (APOL1), PON, vitamin D-binding protein (GC), and SAP) are selectively enriched and Ras-related protein Rab-7b (RAB7B) was reduced in HDL3, while 12 proteins (APOA1, APOE, RAB7B, PON, APOA4, APOL1, HP, HPX, serotransferrin (TF), complement factor B, Fgg, and IGHG1) are decreased and 4 proteins (SAP, SERPINA1, acid ceramidase, and GC) are increased in HDL2. In gestational diabetes mellitus (GDM) patients [45], CEC of maternal GDM-HDL is 25% lower than that of healthy HDL, which is found to be positively correlated with the PON1 activity and contens of APOA1 and APOE. Tomáš Vaisar [46] discovered that in humans, acute inflammation stimulated by endotoxin could result in HDL proteomic changes, with a selective increase in SAA1 and SAA2, which is inversely correlated with impaired HDL CEC from macrophages. In addition to the proteins, the PLs of HDL fractions are strongly correlated with CEC, which can be explained by the fact PLs provide a big sink for cholesterol solubilization [42, 47]. Above all, many proteomic changes in HDL subclasses are found to be involved in altering HDL cholesterol efflux capacity, but there are controversies regarding the HDL protein clusters that determine CEC, in healthy patients or those in disease states.

Meanwhile, the antioxidative property takes part in the cardioprotective effects of HDL [48], and whether the HDL antioxidative capacity is totally determined by HDL proteins is still unclear. Mathew [49] identified that lifestyle changes could enhance HDL function via suppressing oxidation by myeloperoxidase without changes on the proteomic composition in subjects with metabolic syndrome. However, many studies have indicated that alterations in HDL antioxidative capacity are caused by changes to the HDL proteome. Many studies confirm that PON1 and PON3 are the main determinants of the antioxidative function of HDL by influencing oxidative stress through reactive oxygen species (ROS) generation or by preventing the oxidation of LDLs, while other proteins (including lipoprotein-associated phospholipase A2 (Lp-PLA2), APOA1, APOE, LCAT, plasmalogens, TF, Cp, CLU, and platelet-activating factor acetyl hydrolase (PAF-AH)) are also found to account for the antioxidative properties of HDL particles [28, 50–54]. Nevertheless, some researches have discovered that the HDL oxidant index is not associated with PON1 or PON3 activity [45, 55]. Further studies demonstrate the S-HDL [42] contributing to elevated levels of proteins (APOA1, APOA2, APOC1, and CLU) and HDL3 [37, 39] contributing to elevated levels of proteins (PON1, PON3, and APOL1) had enhanced antioxidative capacity in FPLC-purified S/L-HDL subclasses and in UC-purified HDL2/HDL3 subclasses, respectively. Whereas, the present findings revealed M-HDL had a higher antioxidative index to prevent LDL oxidation than the S-HDL subclass, and the relatively abundant proteins played a role in the biological antioxidation were enriched in M-HDL but less in S-HDL, especially in human samples, which is in accordance with the previous study [16] of FPLC-purified S/M/L-HDL subclasses in mouse [16]. However, the particular antioxidative proteins of the HDL subclasses were different. In this study, it was showed that human M-HDL acquired antioxidative functions by recruiting APOE, APOA4, AMBP, Cp, and.catalase (CAT), while rat M-HDL acquired antioxidative functions by recruiting thrombospondin-1 (THBS1), glutathione peroxidase 3 (GPX3), epidermal growth factor receptor (EGFR), and CP. In summary, the different classifications of the HDL subclasses by FPLC or UC and different MS methods may result in different predominant HDL subclasses possessing major antioxidative capacity, as well as various HDL-associated proteins with antioxidative properties even in a specific species.

### Comparisons with other studies and what does the current work add to the existing knowledge

Gordon, S. M [18]. has identified the HDL-associated proteins distribution based on the the peptides across FPLC-purified HDL fraction number (17 subfractions) and the associated roles in the complement regulation and protease inhibition in human, and many studies discover specific proteomic changes in HDL between different diseases and healthy controls. However, little was focusing on the relatively abundant proteins of HDL subclasses. The results of the current work revealed the S-HDL and L-HDL subclasses were likely composed of different proteomic components based on the comparison of the relatively abundant proteins in S/M/L-HDL subclass, which happened to coincide with the biological process of HDL maturation. Moreover, we creatively evaluated the functions of HDL subclasses based on their relatively abundant proteins, which revealed different patterns across S/M/L-HDL subclasses in human and rat.

### Study strengths and limitations

There were two strengths of this research. One was the proteomics comparison of the FPLC purified S/M/L-HDL subclasses performed based on the relatively abundant proteins of each HDL subclass in human and rats, and few studies of protein comparisons between HDL subclasses have conducted in rat. The other was the associated functions based on grouping the relatively abundant proteins in each HDL subclasses were searched and summarized in this study. However, the limitations of this study include as follows. Firstly, while HDL carries various of lipid compositions, including bioactive substances, for example, sphingosine-1-phosphate (S1P) [56], there was a lack of in-depth lipidomics analysis of HDL subclasses. Combined studies on the lipidome and proteome of the HDL subclasses may reveal thorough markers that could be used to interpret the functions of the HDL subclasses. Secondly, due to the three column FPLC system and CSH purification of HDL, the proteins at low concentration, for example APOCs, may be lost or undetectable by MS. And that the particular roles and functions of the relatively abundant proteins still need to be validated. Thirdly, the sample size was limited, especially the *n* = 3 for rats, the findings of HDL subclasses proteomics and functions needed to be further validated in a large-scale study.

## Conclusion

The present proteomic study of HDL subclasses purified by FPLC in healthy human and rat samples discovered that the S-HDL and L-HDL subclasses were likely composed of different proteomic components during HDL maturation based on the relatively abundant proteins analyses, and proteomic comparison of the HDL subclasses may explain the associated differences in function.

The compositions and functions across different HDL particle size are interestingly investigated in clinical trials, focusing on its role in different disease and its clinical benefits. The wide range of the involved diseases includes not only cardiovascular disease (NCT02106013) and metabolic disease (NCT04294238), but also sepsis (NCT02370186), stroke (NCT03245957), cognitive disorder (NCT03761719). In the therapeutic studies of interventions with drugs (Glitazones (NCT00953498), 2-Hydroxybenzylamine (2-HOBA) (NCT04941599), extended-release niacin((NCT00150722)), there are growing concerns about correcting the alterations of the specific HDL compositions and improving the associated effects of HDL subclasses (CEC, antioxidative effect, vasodilatory effect, endothelial function, anti-inflammatory effect, etc.), instead of raising HDL-C level. In addition, the behavioral changes (aerobic exercise (NCT04294238) and dietary supplement (include pomegranate juice and extract, lycopene, and quercetin (NCT04097119)), Vitamin D(NCT02135913) may also improve the quality of life and recovery due to improving HDL composition and function in healthy subjects. The present study demonstrated the proteomic and functional differences base on the relatively abundant proteins across the size of HDL subclasses in healthy subjects, may help to identify efficient strategies for modifying the compositions and associated functions of HDL subclasses.

## Fundings

This study was funded by grants from the National Natural Science Foundation of China (Grant no.: 81970388), Major Fund for International Cooperation in Guangdong Province (Grant no.: 2021A0505030021), the Guangzhou Science and Technology Plan Project (Grant no.: 202102010218), and the Natural Science Foundation of Guangdong Province (Grant no.:2021A1515012359).

## Supplementary Information

**Additional file 1: Table S1.**Mouse HDL particle proteins identified previously in two or more studies. **Table S2**. Acquired mouse and rat HDL proteomic studies information (2005-2021). **Table S3.** Human HDL proteins identified  previously  in three or more studies. **Table S4.** Acquired human  HDL proteomic studies information (2005-2021).

**Additional file 2: Table S5.** The MS data of L/M/S-HDL subclasses from healthy males (*n*=6). **Table S6.**The MS data of L/M/S-HDL subclasses from rats (*n*=3).

## Data Availability

The datasets used and/or analyzed during the current study are available from the corresponding author on reasonable request.

## Abbreviations

S/M/L-HDL: Small, medium, and large high density lipoprotein
HDL: High density lipoprotein;
FPLC: Fast protein liquid chromatography
CSH: Calcium silica hydrate
S-HDL: Small high-density lipoprotein
M-HDL: Medium high-density lipoprotein
L-HDL: Large high-density lipoprotein
HDL-C: High density lipoprotein-cholesterol
miRNAs: MicroRNAs
HPX: Hemopexin
HP: Haptoglobin
SAA: Serum amyloid A
ABCA1: ATP-binding cassette transporter A1
SERPINA1: α1-Antitrypsin
APOC2: Apolipoprotein C-II
PON: Paraoxonase
PON1: Paraoxonase-1
CETP: Cholesteryl ester transfer protein
LDL: Low-density lipoprotein
SD: Sprague–Dawley
EDTA: Ethylene diamine tetraacetic acid
PLs: Phospholipids
LC–MS/MS: Liquid chromatography-mass spectrometry/mass spectrometry
MS: Mass spectrometry
LFQ: Label-free quantification
GO: Gene Ontology
CEC: Cholesterol efflux capacity
DMEM: Dulbecco’s Modified Eagle’s Medium
FBS: Fetal bovine serum
ACAT: Sterol O-acyltransferase 1
cAMP: Cyclic adenosine monophosphate
BSA: Bovine serum albumin
PBS: Phosphate-buffered saline
ox-LDL: Oxidized low-density lipoprotein
DHR: Dihydrorhodamine 123
SDS-PAGE: Sodium dodecyl sulfate–polyacrylamide gel electrophoresis
APO: Apolipoprotein
APOA1: Apolipoprotein A-I
ALB: Albumin
IGLL5: Immunoglobulin lambda-like polypeptide 5
CLU: Clusterin
SAA4: Serum amyloid A4
APOA2: Apolipoprotein
KNG1: Kininogen 1
IGKV3-20: Immunoglobulin kappa variable 3–20
CRTAC1: Cartilage acidic protein 1
Proz: Vitamin K-dependent protein Z
LOC299282: Serine protease inhibitor
IGH-1a: Immunoglobulin gamma-2B chain C region
F13b: Coagulation factor XIII B chain
HBA1: Hemoglobin subunit alpha-1
A2M: Alpha-2-Macroglobulin
ITGA2: Integrin alpha-2
A1BG: Alpha-1-beta glycoprotein
APBA1: Amyloid-beta A4 precursor protein-binding family A member 1
B2M: β2-Microglobulin
AMBP: Alpha-1-microglobulin/bikunin precursor
CST3: Cystatin-C
CFD: Complement factor D
RBP4: Retinol-binding protein 4
PON3: Paraoxonase-3
LCAT: Lecithin cholesterol acyl transferase
UC: Ultracentrifugation
SEC: Size-exclusion chromatography
APOC3: Apolipoprotein C- III
LBP: Lipopolysaccharide-binding protein
APOE: Apolipoprotein E
LPA: Apolipoprotein (a)
APOA2: Apolipoprotein A-II
APOD: Apolipoprotein D
PLTP: Phospholipid transfer protein
APOA4: Apolipoprotein A –IV
APOC1: Apolipoprotein C –I
ANT3: Antithrombin-III
SAP: Serum amyloid P
A2AP: Alpha-2-antiplasmin
GELS: Macrophage-capping protein
IGHG1: Immunoglobulin heavy constant gamma 1
Cp: Ceruloplasmin
ACS: Acute coronary
APOL1: Apolipoprotein L1
GC: Vitamin D-binding protein
Fgg: Fibrinogen gamma chain
RAB7B: Ras-related protein Rab-7b
TF: Serotransferrin
GDM: Gestational diabetes mellitus
ROS: Reactive oxygen species
Lp-PLA2: Lipoprotein associated phospholipase A2
PAF-AH: Platelet-activating factor acetylhydrolase
CAT: Catalase
THBS1: Thrombospondin-1
GPX3: Glutathione peroxidase 3
EGFR: Epidermal growth factor receptor
APOM: Apolipoprotein M
S1P: Sphingosine-1-phosphate
